# A New Quantitative Method for Assessing the Ultrastructure of Cardiomyocyte Mitochondria of the Right Atrial Appendage

**DOI:** 10.17691/stm2026.18.2.05

**Published:** 2026-04-30

**Authors:** E.A. Kuzheleva, A.A. Garganeeva, O.V. Tukish, E.E. Syromyatnikova, B.B. Dorzhieva, M.Yu. Kondratiev, S.L. Andreev

**Affiliations:** MD, PhD, Senior Researcher, Department of Myocardial Pathology; Cardiology Research Institute, Tomsk National Research Medical Center of the Russian Academy of Sciences, 111a Kievskaya St., Tomsk, 634012, Russia; MD, DSc, Professor, Head of the Department of Myocardial Pathology; Cardiology Research Institute, Tomsk National Research Medical Center of the Russian Academy of Sciences, 111a Kievskaya St., Tomsk, 634012, Russia; MD, PhD, Researcher, Department of Myocardial Pathology; Cardiology Research Institute, Tomsk National Research Medical Center of the Russian Academy of Sciences, 111a Kievskaya St., Tomsk, 634012, Russia; Resident Physician, Department of Myocardial Pathology; Cardiology Research Institute, Tomsk National Research Medical Center of the Russian Academy of Sciences, 111a Kievskaya St., Tomsk, 634012, Russia; Resident Physician, Department of Myocardial Pathology; Cardiology Research Institute, Tomsk National Research Medical Center of the Russian Academy of Sciences, 111a Kievskaya St., Tomsk, 634012, Russia; Junior Researcher, Department of Myocardial Pathology; Cardiology Research Institute, Tomsk National Research Medical Center of the Russian Academy of Sciences, 111a Kievskaya St., Tomsk, 634012, Russia; MD, PhD, Senior Researcher, Department of Cardiovascular Surgery; Cardiology Research Institute, Tomsk National Research Medical Center of the Russian Academy of Sciences, 111a Kievskaya St., Tomsk, 634012, Russia

**Keywords:** chronic heart failure, atrial fibrillation, mitochondrial ultrastructure, electron microscopy, total area of interfibrillar mitochondria

## Abstract

**The aim of this study** was to develop a quantitative method for assessing the ultrastructure of cardiomyocyte mitochondria in the right atrial appendage of patients with chronic heart failure (CHF).

**Materials and Methods:**

A single-center prospective study included 39 patients aged 67 [58; 71] years who underwent coronary artery bypass grafting. The inclusion criteria were as follows: heart failure with a left ventricular ejection fraction of <50%, atherosclerotic plaques in two or more large coronary arteries of 70% or greater, and the cardiac team’s decision to perform coronary artery bypass grafting. To perform electron microscopy, biopsy samples were collected from the right atrial appendage during coronary artery bypass grafting. Two calculated parameters were used for the analysis: “total area of interfibrillar mitochondria” and “ratio of outer to inner membrane length”. Based on these, the total index was calculated by the formula: the “total area of interfibrillar mitochondria” / the “ratio of outer to inner membrane length”.

**Results:**

The median values of the calculated parameters characterizing the ultrastructure of cardiomyocyte mitochondria in the right atrial appendage were as follows: 43.7 [35.9; 54.3]% for the “total area of interfibrillar mitochondria”, 31 [25; 37]% for the “ratio of outer to inner membrane length”, and 1.4 [0.95; 2.00] for the total index accounting for both ultrastructural characteristics of mitochondria: the “total area of interfibrillar mitochondria” / the “ratio of outer to inner membrane length”.

To assess the clinical significance of the proposed total index, associations between the obtained values and atrial fibrillation (AF) were studied. Mitochondrial ultrastructural changes were more pronounced in patients with both AF and CHF. However, some parameters had no statistically significant differences: the total area of interfibrillar mitochondria was lower in patients with AF (42% vs 49%, p=0.224), and the ratio of outer to inner membrane length was higher in patients with AF (35% vs 31%, p=0.125). The total index was significantly lower in patients with AF (0.96 vs 1.75, p=0.021). ROC analysis was performed to identify a connection between total index and AF, and AUC was 0.773 (p=0.021).

**Conclusion:**

The proposed total index for analyzing cardiomyocyte micrographs, calculated as the “total area of interfibrillar mitochondria” / the “ratio of outer to inner membrane length”, provides a comprehensive characterization of mitochondria that accounts not only for their number and size but also for their internal structure.

## Introduction

Chronic heart failure (CHF) is the final stage of the cardiovascular continuum and is a major challenge in modern medicine [1]. To date, there has been a significant amount of experimental and clinical data accumulated showing a close association between CHF development and pathological changes in cardiomyocyte mitochondria structure and function. The development of drug molecules targeting mitochondrial pathways is considered promising for optimizing pharmacological therapy for heart failure [2].

According to modern concepts, mitochondrial function is closely associated with its structural state [3]. However, assessing the ultrastructure of cardiomyocyte mitochondria is extremely challenging due to the need to collect myocardial biopsy samples. This is connected with complications and not justified by the ethical standards of scientific research. A potential solution could be collecting myocardial biopsies from the right atrial appendage during cardiac surgery with cardiopulmonary bypass. The tissue collection is performed during the stage of right atrial cannulation, which prevents additional injury to the heart muscle.

An additional difficulty is the interpretation of micrographs of cardiomyocyte mitochondria. Currently, there is no universally accepted and validated method for quantitative assessment of the ultrastructure of cardiomyocyte mitochondria. An optimal method for assessing the ultrastructure of the mitochondria in CHF should be reproducible and pathomorphologically valid. The method should also ensure a correlation between the results of assessment of cardiomyocyte micrographs and clinical data that characterize the severity of heart failure signs, such as systolic and/or diastolic parameters, exercise tolerance, and functional class of heart failure.

Some publications propose methods for the quantitative assessment of mitochondrial ultrastructure. For example, for left ventricular cardiomyocytes, quantitative parameters describing mitochondrial density were described, representing the ratio of their area to the area of the cardiomyocyte or the area of myofibrils [4]. However, first, the presented method was developed for assessing mitochondria in laboratory animals, not humans. Secondly, these parameters are practically inapplicable for assessing cardiomyocyte mitochondria in the right atrial appendage due to poorly structured cell arrangements in this tissue. Previously, we proposed a calculated parameter, “total area of interfibrillar mitochondria” in right atrial appendages cardiomyocytes defined as the ratio of total area of interfibrillar mitochondria to area of interfibrillar space [5]. This calculated parameter demonstrated clinical relevance, as the obtained values correlated with exercise tolerance in CHF patients, assessed by the six-minute walk test, and with peak oxygen consumption during exercise, measured by spiroergometry [5]. However, this previously proposed method does not provide a complete understanding of the morphology of cardiomyocyte mitochondria, as it does not account for their internal structure.

To assess the internal structure of mitochondria, El’darov et al. [6] proposed a method for calculating the ratio of inner membrane surface area to mitochondrial volume, which was also developed and applied to assess ultrastructural changes in cardiomyocyte mitochondria of certain laboratory rat strains, not humans. This method is complex, as it requires preliminary calculations of area and volume, and does not take into account the number of mitochondria or their density between myofibrils.

Thus, to date, there is no comprehensive method for the quantitative assessment of the ultrastructure of human cardiomyocyte mitochondria in the right atrial appendage.

**The aim of this study** was to develop a quantitative method for assessing the ultrastructure of cardiomyocyte mitochondria in the right atrial appendage of patients with chronic heart failure.

## Materials and Methods

This single-center prospective study included 39 patients aged 67 [58; 71] years who underwent coronary artery bypass grafting (protocol registered at ClinicalTrials.gov: NCT05770349). The study protocol adhered to the principles of the Declaration of Helsinki (2024) and was approved by the local ethics committee of the Cardiology Research Institute, Tomsk National Research Medical Center of the Russian Academy of Sciences (Russia) (protocol No.241 dated March 9, 2023).

The study design was described in detail in our previous publications [5, 7]. The inclusion criteria were as follows: heart failure with left ventricular ejection fraction of <50%, atherosclerotic plaques in two or more large coronary arteries of 70% or greater, the cardiac team’s decision to perform coronary artery bypass grafting, and signed informed consent from the patient to participate in the study. Patients were excluded from the study in the following cases: refusal of revascularization, need for additional cardiac surgery other than coronary artery bypass grafting, active oncological disease or implanted devices, severe renal dysfunction (estimated glomerular filtration rate CKD-EPI <30 ml/min/1.73 m^2^), infiltrative heart disease, acute infection or exacerbation of chronic diseases, severe chronic obstructive pulmonary disease, bronchial asthma, any type of diabetes mellitus, or anemia.

The collected information included patient complaints, medical history, standard laboratory and instrumental examinations of all patients, including echocardiography, measurement of N-terminal pro-brain natriuretic peptide levels. Biopsy samples were collected from the right atrial appendage during coronary artery bypass grafting. Electron microscopy was performed using a JEM-1400 transmission electron microscope (JEOL Ltd., Japan). Image processing was carried out using ImageJ software [5].

**Figure 1.**
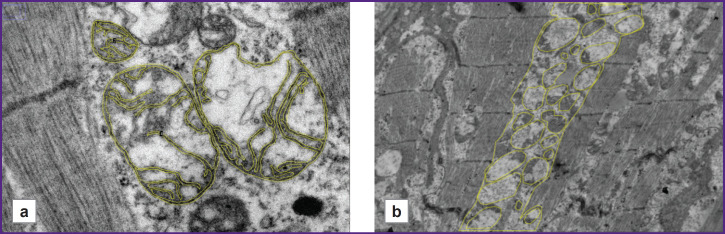
Micrographs of a right atrial appendage cardiomyocyte: (a) 15,000×; the outer and inner mitochondrial membranes are outlined with a yellow line; (b) 5000×; the contour of the interfibrillar space and the mitochondria located inside it are outlined with a yellow line

On the micrographs at 15,000× magnification (Figure 1 (a)), the ratio of outer mitochondrial membrane length to inner mitochondrial membrane length was calculated. Three micrographs were analyzed for each patient. For each micrograph, this parameter was calculated for three mitochondria. The result was calculated as the arithmetic mean; the obtained value was expressed as a percentage.

On micrographs at 5000× magnification (Figure 1 (b)), the total area of interfibrillar mitochondria was calculated as the ratio of the total area occupied by mitochondria located between cardiomyocyte contractile fibers and the total area in the interfibrillar space. The obtained value was expressed as a percentage [5].

Then, a total index accounting for both the ultrastructural characteristics of mitochondria (considering their opposite directionality) was calculated using the formula: the “total area of interfibrillar mitochondria” / the “ratio of outer membrane length to inner membrane length”.

***Statistical analysis of the results*** was performed using IBM SPSS 21.0 software. Quantitative continuous variables were presented as the median and interquartile range — Me [Q1; Q3]. Categorical data was presented as absolute and relative frequencies — n (%). Continuous variables in independent samples were analyzed using the Mann–Whitney test. To determine the connection between the presence of atrial fibrillation (AF) and the values of quantitative mitochondrial ultrastructure parameters, ROC analysis was used. Differences were considered statistically significant at p≤0.05.

## Results

The median values of the calculated parameters characterizing the ultrastructure of cardiomyocyte mitochondria in the right atrial appendage were as follows: the “total area of interfibrillar mitochondria” was 43.7 [35.9; 54.3]%; the “ratio of outer to inner membrane length” was 31 [25; 37]% and the total index accounting for both ultrastructural characteristics of mitochondria, calculated as the “total area of interfibrillar mitochondria” / the “ratio of outer membrane length to inner membrane length”, was 1.4 [0.95; 2.00].

To assess the clinical significance of the proposed total index, associations between the obtained values and the presence of AF were studied.

AF was diagnosed in 12 (30.8%) patients in the study group. Based on the presence or absence of AF, the patients were divided into two groups: group 1 consisted of patients without AF (n=27), and group 2 consisted of patients with AF (n=12).

**Figure 2.**
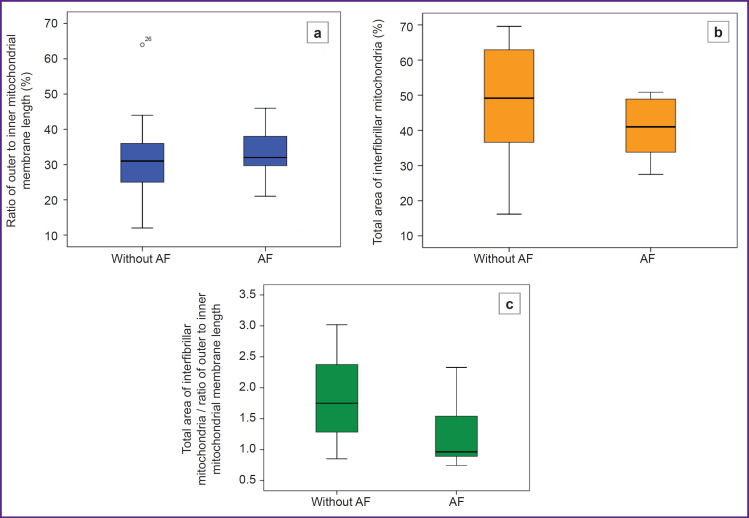
Comparison of the parameters of the cardiomyocyte mitochondrial ultrastructure in patient groups without and with atrial fibrillation (AF) (a) parameter characterizing mitochondrial density (p=0.125); (b) parameter characterizing the internal mitochondrial structure (p=0.224); (c) value of the total index (p=0.021)

The individual parameters characterizing the mitochondrial density and their internal structure did not differ significantly between the patient groups. The “total area of interfibrillar mitochondria” was slightly lower in patients with AF and amounted to 42 [33; 49]%, whereas in patients without AF it was 49 [36; 64]% (p=0.224) (Figure 2 (b)). The “ratio of outer to inner membrane length”, was also slightly lower in patients without AF, suggesting a more preserved internal mitochondrial structure. This is supported by the fact that the total index in this group compared to patients with AF (31 [25; 36]% vs 35 [30; 45]%, respectively; p=0.125) (Figure 2 (a)).

Then, the total index accounting for both ultrastructural characteristics of mitochondria was calculated. The result was statistically significant (p=0.021) with a value of 1.75 [1.28; 2.39] in patients without AF and 0.96 [0.89; 1.54] in patients with AF (Figure 2 (c)).

**Figure 3.**
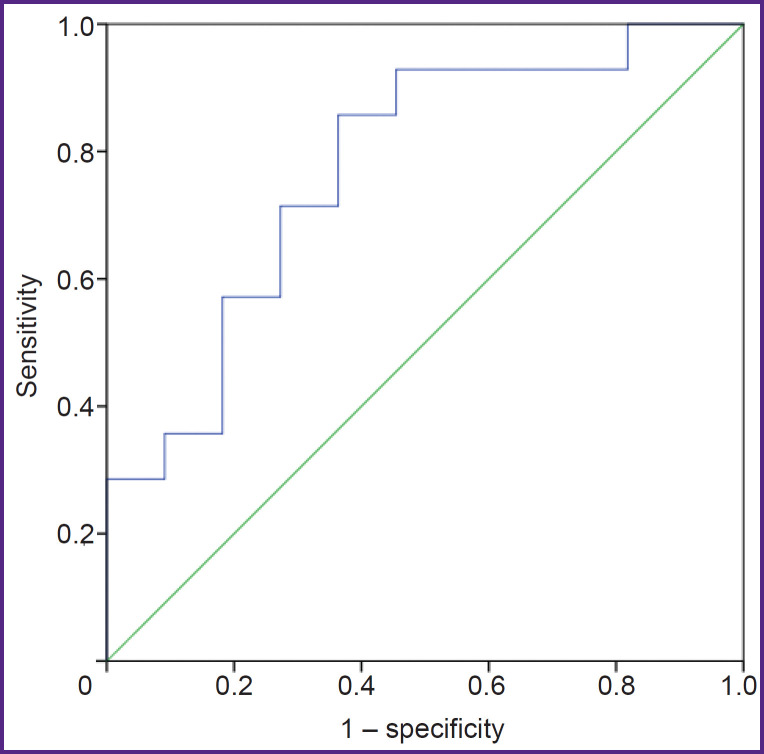
ROC analysis identifying the connection between the total index calculated as the “total area of interfibrillar mitochondria” / the “ratio of outer to inner mitochondrial membrane length” and the presence of atrial fibrillation The area under the ROC curve (AUC) was 0.773; p=0.021

ROC analysis was also performed to identify the connection between the total index calculated as the “total area of intermyofibrillar mitochondria” / the “ratio of outer to inner membrane length” and the presence of AF (Figure 3). The area under the ROC curve (AUC) was 0.773 (p=0.021).

**Figure 4.**
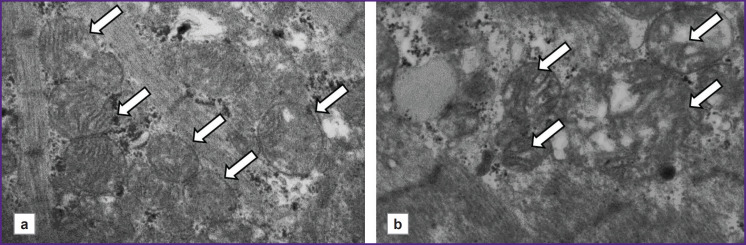
Representative micrographs of cardiomyocytes at 15,000× magnification The shown micrographs are from patients with chronic heart failure with reduced ejection fraction: (a) with atrial fibrillation (left ventricular ejection fraction — 28%); (b) without atrial fibrillation (left ventricular ejection fraction — 37%); arrows indicate interfibrillar mitochondria

Figure 4 shows micrographs illustrating differences in the ultrastructure of cardiomyocyte mitochondria in patients with CHF depending on the presence or absence of AF. It should be noted that, in a patient without AF, the mitochondria are closely adjacent to one another, and their internal structure is visualized more distinctly. In contrast, in the patients with AF, mitochondrial shapes and sizes vary, and they are separated from one another. In some mitochondria, cristae have been disrupted.

## Discussion

Quantitative assessment of cardiomyocyte mitochondrial ultrastructure in CHF is an extremely important and promising medical research area. The concept of supranosological therapy is currently being developed. It aims to correct basic pathophysiological processes leading to multiple beneficial effects in different diseases. In particular, this applies to one of the major medication classes for CHF — sodium-glucose cotransporter 2 inhibitors (SGLT2i). This medication was originally developed to treat diabetes mellitus but has shown favorable effects on CHF clinical course and outcomes regardless of left ventricular ejection fraction [1, 8]. The impact of SGLT2i on mitochondrial structure and function is currently actively discussed as one of mechanisms underlying its cardioprotective effects [2, 9, 10]. However, without an effective method for the quantitative assessment of cardiomyocyte mitochondrial ultrastructure, it is impossible to objectively characterize the state of these organelles and, consequently, to evaluate the efficacy of specific drug classes in improving mitochondrial structural and functional parameters.

The calculated parameters proposed in our study, such as the “total area of interfibrillar mitochondria” [5] and the “ratio of outer to inner mitochondrial membrane length”, enable the characterization of various aspects of the ultrastructural organization of cardiomyocyte mitochondria, such as the density of these organelles between myofibrils and the integrity of cristae which is necessary for normal functioning.

Similar methods for the quantitative assessment of mitochondrial ultrastructure have been previously proposed. Specifically, to assess the density and number of mitochondria in laboratory animal cardiomyocytes, the ratio of mitochondrial area to cardiomyocyte or myofibril area has been used [4]. To assess the internal structure of mitochondria in laboratory animal cardiomyocytes, a calculated parameter, defined as the ratio between the inner membrane surface area and mitochondrial volume, has been proposed [6].

For the quantitative assessment of mitochondrial ultrastructure, Hayashi et al. [11] proposed a method for calculating a mitochondria damage index based on determining the severity of the degeneration of cristae. The calculation method was difficult, and the results were subjective because the authors had to count the mitochondria in the same field of view six times and average the results.

However, none of the previously proposed methods [4, 6, 11] for the quantitative assessment of mitochondria have simultaneously accounted for multiple characteristics, such as the density and integrity of the internal structure of mitochondria. Thus, we propose to combine the obtained values into a single total index, calculated as the “total area of interfibrillar mitochondria” / the “ratio of outer to inner membrane length”. This index provides a comprehensive characterization of mitochondria, considering not only their number and size but also their internal structure.

To confirm clinical significance, the values of calculated parameters were compared between patient groups with and without AF.

It is well known that AF and CHF negatively influence each other’s pathogenesis and patient prognosis [12–14]. Previous studies have clearly demonstrated the signs of structural remodeling of atrial myocardium in AF, including fibrosis, lipomatosis, isolated atrial amyloidosis, and cardiomyocyte hypertrophy with partial myofibril loss [15]. Experimental studies have also shown structural changes of atrial cardiomyocyte mitochondria in patients with AF [16, 17].

Therefore, it was important to confirm the clinical relevance of the newly proposed total index, calculated as the “total area of interfibrillar mitochondria” / the “ratio of the outer to the inner membrane length”. The results are consistent with the literature data: AF certainly has an additional negative impact on mitochondrial ultrastructure in patients with severe CHF of ischemic etiology. This is reflected in statistically significant differences in the total index between patients with and without AF. These findings have not been previously described in the literature before; they allow a deeper understanding of negative associations between these conditions from clinical and prognostic perspectives.

It should be noted that initially, the studied patient cohort had severe heart failure, with reduced left ventricular ejection, fraction and multivessel stenosing coronary atherosclerosis. At the ultramicroscopic level, this was associated with structural changes in cardiomyocyte mitochondria [18]. Additionally, AF led to a reduction in the number of mitochondria between cardiomyocyte myofibrils in the right atrial appendage, as well as a decrease in the number of inner mitochondrial membrane cristae. These changes undoubtedly affected the functional efficiency of these organelles.

### Study limitations

The main limitation was the relatively small sample size of only 39 patients but for studies with similar design involving electron microscopy of human myocardium, this sample size was substantial, especially given that over 250 micrographs were analyzed during processing of the electron microscopy results.

## Conclusion

The proposed total index for analyzing cardiomyocyte micrographs, calculated as the “total area of interfibrillar mitochondria” / the “ratio of outer to inner membrane length”, allows a comprehensive characterization of mitochondria, taking into account not only their number and size but also their internal structure.
